# Proposal of Characterization Procedure of Metal–Graphite Interface Strength in Compacted Graphite Iron

**DOI:** 10.3390/ma11071159

**Published:** 2018-07-07

**Authors:** Edwin A. Lopez-Covaleda, Sepideh Ghodrat, Leo A.I. Kestens, Charles-Henry Sacre, Thomas Pardoen

**Affiliations:** 1 Metals Science and Technology Group, EEMMeCS Dept., Ghent University; Technologiepark 903, 9052 Gent, Belgium; Leo.Kestens@UGent.be; 2 Department of Materials Science and Engineering, Delft University of Technology; Mekelweg 2, 2628 CD Delft, The Netherlands; S.Ghodrat@tudelft.nl; 3 Institute of Mechanics, Materials and Civil Engineering (IMMC), Université Catholique de Louvain; Réaumur, Place Sainte Barbe 2, B-1348 Louvain-la-Neuve, Belgium; charles-henry.sacre@uclouvain.be (C.S.); thomas.pardoen@uclouvain.be (T.P.)

**Keywords:** bonding strength, scratch test, compacted graphite iron

## Abstract

Compacted graphite iron is the material of choice for engine cylinder heads of heavy-duty trucks. Compacted graphite iron provides the best possible compromise between optimum mechanical properties, compared to flake graphite iron, and optimum thermal conductivity, compared to spheroidal graphite iron. The vermicular-shaped graphite particles, however, act as stress concentrators, and, as a result of delamination from the metal matrix, they are responsible for crack initiation during the thermomechanical fatigue cycles occurring through engine startup and shutdown cycles. Scratch tests driven over the matrix and into the graphite particles were performed in order to characterize the strength of the metal–graphite interface. Samples extracted from a cylinder head in as-cast condition were compared to samples subjected to a heat-treatment at 700 °C for 60 h. The former samples were composed of a primarily pearlitic matrix and graphite particles (~11.5 vol %), whereas, after annealing, a certain pearlite fraction decomposed into Fe and C, producing a microstructure with graphite–ferrite interfaces, exhibiting a partially spiky morphology. The scratch test revealed that the ferrite–graphite interfaces with spiky nature exhibited a stronger resistance to delamination compared to the ferrite–graphite interfaces with smooth morphology. One reason for the high interface strength is the mechanical interlocking between graphite spikes and ferrite, increasing the contact area between the two phases.

## 1. Introduction

Compacted graphite iron (CGI) is the material of choice for manufacturing cylinder heads of heavy trucks. Since these cylinder heads are subject to cyclic thermal and mechanical loads in daily startup and shutdown cycles, the CGI component is subjected to thermomechanical fatigue (TMF). There is a growing interest from truck manufacturers in modeling TMF behavior with microstructurally based material descriptors. Current finite element (FE) simulations of the mechanical behavior of cast iron usually neglect the bonding between the graphite particles and the metal matrix [1,2,3,4,5,6], because there is a lack of knowledge about the magnitude of the interface toughness due to the technical difficulty of measuring this property. Therefore, if one would succeed in measuring the bond strength in a quantitative and reliable manner, the accuracy of such models could be drastically improved. The previous work of Ghodrat [1] showed that the TMF behavior of CGI is largely determined by the distribution and morphology of the compacted graphite particles; the former determines the crack propagation rate, and the latter the resistance to crack initiation. However, the critical conditions for crack initiation, arrest, and propagation are not known. A number of researchers have considered the possibility of molecular bonding as primary source of adhesive strength at the metal–graphite (M–G) interface [7,8]. He et al. [9] and Lundberg et al. [10] have observed that, during in situ tensile testing of flake graphite iron, the first cracks occurred at the M–G interface of graphite particles, with their long axis perpendicular to the main loading direction, at a strain of ε = 0.03%. They also noticed that particles exhibiting de-cohesion at the interface were not necessarily the sharpest ones. 

Ghodrat et al. [11] performed TMF tests on CGI extracted from non-used cylinder heads, and annealed samples (720 h at 420 °C under atmospheric conditions) simulating the engine heat load. It was found that fatigue lifetime at room temperature of annealed samples was increased by 300%. Ghodrat et al. concluded that the growth of spikes around some graphite particles (induced by decomposition of pearlite into ferrite and graphite during the heat treatment), may have delayed the crack initiation due to an increase of the interface resistance.

One approach to directly monitor the bond strength is by carrying out scratch tests crossing the M–G interface. The intent of a scratch test is to indent the sample surface in load-controlled mode using nano- or micro-indenting equipment, while the sample is displaced horizontally. During the test, both the lateral force (parallel to the displacement of the indenter), and the normal force can be recorded; additionally, the dimensions and the morphology of the scratch can be analyzed. Ryut [12] studied the failure mode (cutting, wedge, and ploughing) and lateral force relationship with the applied normal force when flake graphite iron was scratched with a sharp indenter. During the test, graphite flakes were removed, and characteristic patterns of friction coefficient were linked to failure modes. Alternatively, Waudby et al. [13], using a Rockwell B sphere, performed scratch tests on nodular cast iron. In this work, the nodules were not removed from the pearlitic matrix. It was also observed that the friction coefficient was reduced, and circumferential cracks developed around the particles. The failure modes are in accordance with simulations made by several researchers [14,15,16]. However, the models are focused on continuous and homogeneous materials free of defects, whereby the stress field in front of the tool has not been reported, nor the interaction with weaker particles, such as graphite.

**Figure 1:**
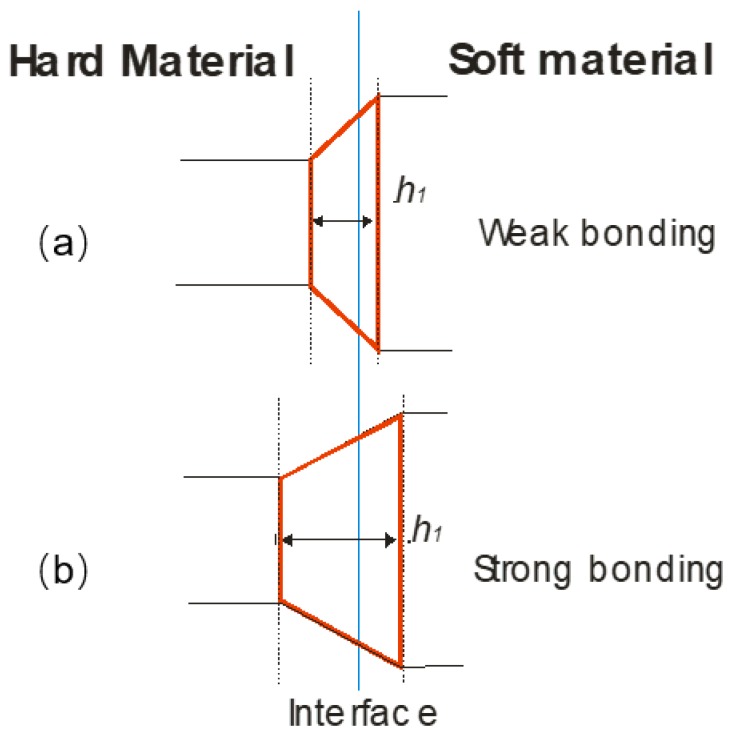
Top view of the trapezium shaped transition area created by a moving indenter when crossing the interface between a hard and a soft phase; it appears that the height *h* of the trapezium increases with bond strength: (**a**) weak bonding and (**b**) strong bonding, respectively.

In a scratch test, the interface is characterized by observing the scratch path transition at the crossing of substrate to coatings, where the crack shape transition zone resembles a trapezium [17] (cf. Figure 1). The link between the trapezium shape and the bond strength was initially investigated in the coatings industry, where it is critical to evaluate adhesive and cohesive bonding. Starting in 1997, Bianchi et al. [18] and later, other authors such as Lin et al. [19] and Erickson et al. [20], performed scratch tests on the transverse section of coated and non-coated thermally sprayed deposits. The scratch on the coated sections started in the substrate, reaching the coating, and finally, the free surface, while in the uncoated substrates, the scratch path went from the bulk to the free surface. They concluded that the transition geometry, characterized by the height (*h*) of the trapezium at the interface, is related to the matrix toughness, and that the reduction of the height is related with improvement of toughness [21]. Moreover, Vencl et al. [17] linked the trapezium height *h* to the bonding strength and interface toughness by carrying out scratch tests on the interface bulk-coating in the transversal section of plasma coatings. The characteristic morphology transition has been modeled based on cutting theory, which proposes a crack propagating in front of the tool edge (in this case, the indenter). Simulations [22,23] and experimental work [24] are mainly focused on applying constant or incremental loads on the indenter scratching over the coated surface because sample preparation of transversal sections may involve early delamination. 

Equivalently, scratch tests applied to evaluate bonding of thermal spray coatings, was used at the M–G interfaces. The trapezium height from metal matrix toward graphite contains mixed information about the mechanical properties of the neighboring phases and of the strength of the interface. Therefore, it is reasonable to assume that the trapezium height *h* is a function of the difference in materials hardness (ΔH) and of the bonding strength of the interface, which can be quantified as the critical stress σ_c_, normal to the interface, which is required to delaminate the two phases (cf. Equation (1)),

*h* = f(ΔH,σ_c_) (1)

In the present study, the scratch test approach was used to characterize the M–G interface. The testing parameters were carefully selected, the force data were recorded, and scratched surfaces were analyzed, trying to quantify the characteristics of the interface. The knowledge acquired from these tests was used to understand the enhancement in mechanical properties after annealing of the cast iron, e.g., a 300% lifetime increase in a low-cycle fatigue test was reported by Ghodrat et al. [11]

## 2. Materials and Methods 

**Figure 2:**
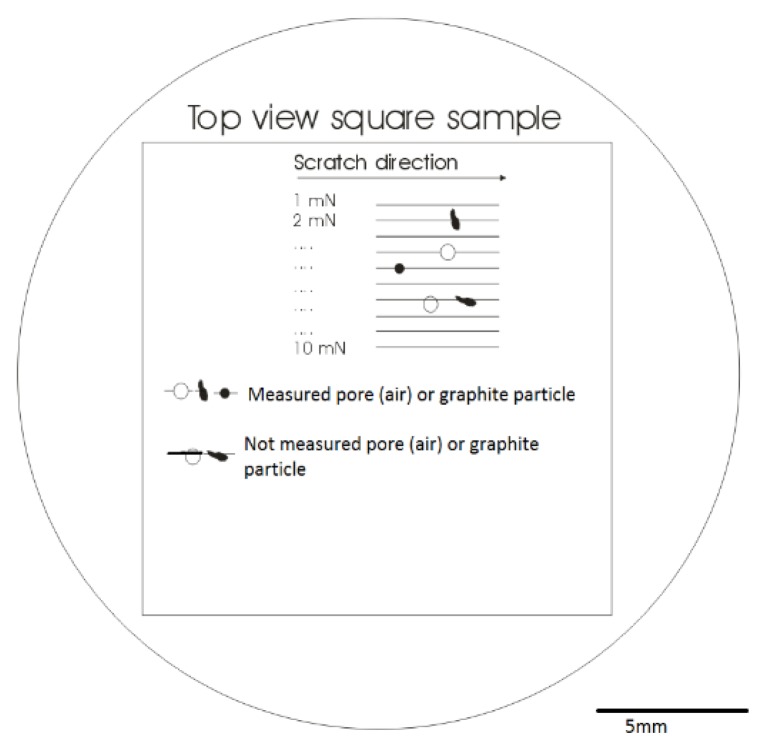
Schematic view of the embedded sample used for the scratch tests, indicating the scratches at metal/graphite and metal/porosity (air) interfaces.

**Figure 3:**
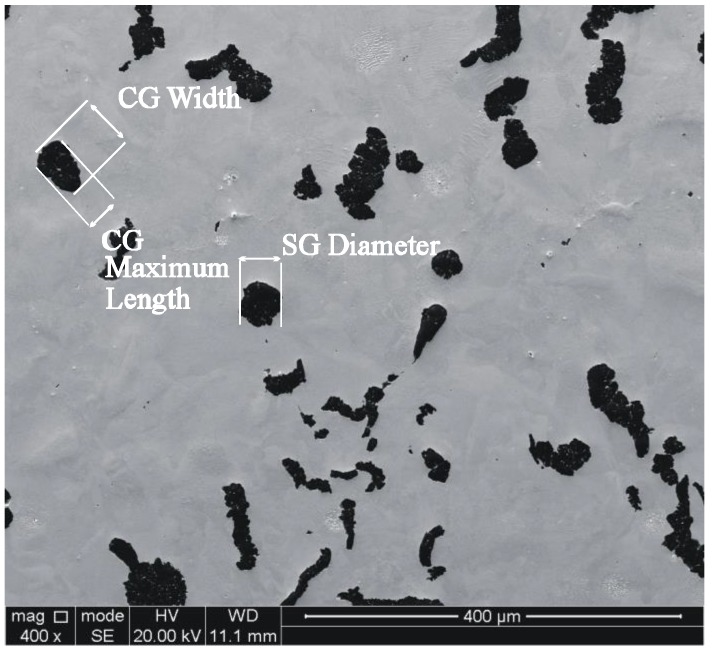
Microstructure of compacted graphite CG and spheroidal graphite in CGI in as-cast (AC) condition.

**Figure 4:**
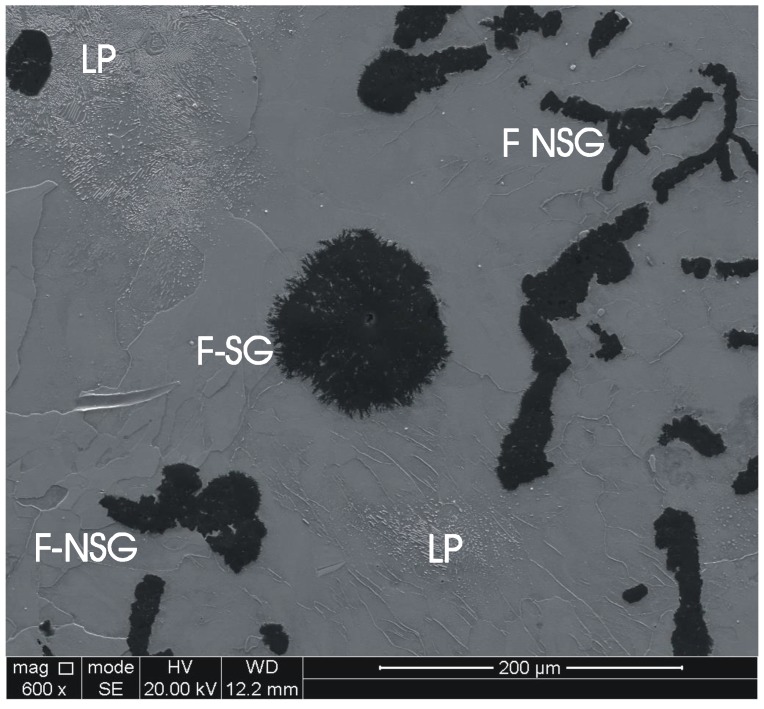
Heat-treated sample with lamellar pearlite (LP), spiky graphite particles (SG), non-spiky graphite (NSG), and decomposed lamellar pearlite to ferrite (F).

Two samples of pearlitic CGI (2 × 2 × 2 cm^3^) (schematic top view in Figure 2) were extracted from the same area of a non-used cylinder head of a truck engine. The first sample corresponds to the as-cast (AC) condition (hardness 258HVN) with 4.5% ferrite, 11.5% compacted graphite, exhibiting the typical vermicular morphology, and pearlite by balance. The characteristics of the graphite particles are listed in Table 1, see also Figure 3. The second sample was heat treated (HT) under atmospheric condition for 60 h at 700 °C. The aim of this heat treatment was to accelerate the decomposition of pearlite into ferrite and graphite, see Figure 4.

After embedding the samples in hard bakelite, they were ground and polished, successively, with 3 and 1 μm diamond suspension paste. The last step was 45 min polishing with 0.5 colloidal silica to provide a flat and smooth surface free from residual stress. Scratch tests were performed using an Agilent Technologies Nano Indenter G200^®^ (Agilent Technologies, Palo Alto, CA, USA). In order to constrain the plastic deformation in front of the indenter, the scratch speed was limited to 20 μm/s [25]. In addition, the cutting-edge angle was reduced using a conic diamond indenter of 60° apex angle and a 1 μm tip radius [21,25]. Furthermore, scratches were applied with 10 constant normal loads varying from 1.0 mN to 10 mN, along 5 mm length lines on the polished AC and HT samples (see Figure 2) crossing dozens of M/G or M/Air interfaces. After the test, the tracks left by the indenter were observed by Scanning Electron Microscope (SEM) (of type FEI Quanta 450^®^ with field emission gun filament, FEI, Portland, OR, USA). At the interface of the metal matrix with a graphite particle or with a porosity, the scratch left a trapezium-shaped trace under the condition that the scratch path was perpendicular to the interface. For both AC and HT samples, the height *h* of this trapezium was measured on the secondary electron images with a magnification of 15,000 to 20,000. In addition, electron-dispersive X-ray (EDAX, Mahwah, NJ, USA) spectroscopy was employed to determine the effect of the heat treatment on the distribution of chemical elements distribution and pearlite decomposition.

## 3. Results

As a result of the heat treatment, three different microstructure elements were obtained (see Figure 4): untransformed lamellar pearlite (labeled LP, see Figure 4) with Vickers hardness HVN = 234, ferrite (labeled F) with Vickers hardness 133HVN, adjacent to either spiky graphite (labeled SG) or unmodified graphite (labeled NSG). Table 2 lists the chemical element composition of the metal matrix in the as-cast and HT samples, whereby, for the HT condition, a separate elemental analysis was made for the ferrite and pearlite phases. The last row of Table 2 indicates the chemical composition of a very similar material investigated by Ghodrat [1]. As this composition was measured by the high-frequency induction furnace LECO CS-225 (LECO, St. Joseph, MI, USA) and an ARL™ PERFORM’X X-ray fluorescence analyzer (XRF) (Thermo Scientific, Waltham, MA, USA) technique, the carbon content could also be determined.

**Figure 5:**
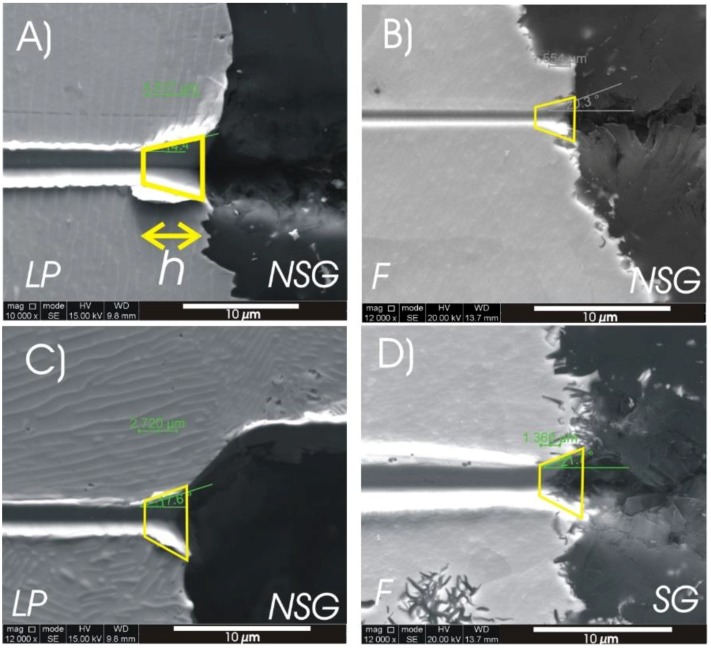
Trapezium shaped track transition area in as-cast (**A**) and heat-treated samples (**B**–**D**) between (**A**) lamellar pearlite and smooth graphite (LP/NSG); (**B**) ferrite and non-spiky graphite (F/NSG); (**C**) lamellar pearlite and non-spiky graphite; and (**D**) ferrite and spiky graphite (F/SG).

**Figure 6:**
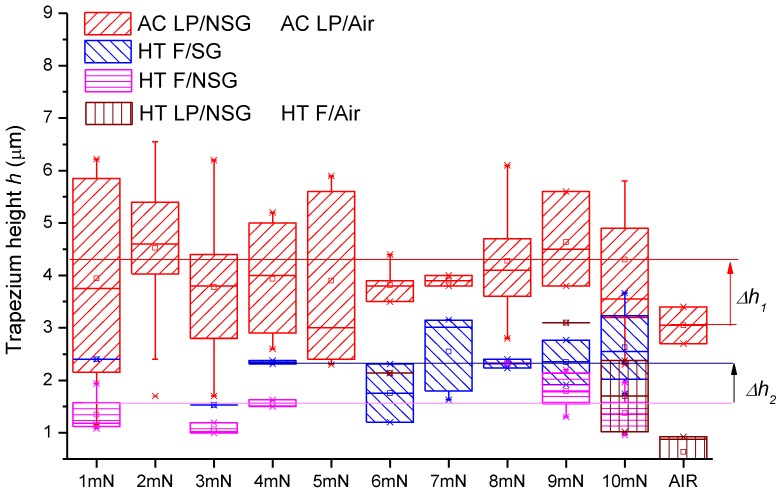
Trapezium height measured in scratched samples, at least 5 measurements were performed in each category. The box height corresponds to 50% of the data around the median value indicated by the symbol □. The range indicated by the symbol * expands over ±1.5 times STDEV.

Figure 5 shows the SEM secondary electron image of the track transition from the matrix phase to a graphite particle (from left to right) as observed on the AC sample (see Figure 5A) and on the HT samples (see Figure 5B–D). On these micrographs, the lamellar pearlite, ferrite, spiky graphite, and non-spiky graphite phases are indicated by LP, F, SG, and NSG, respectively. At the M–G interface, the scratch trace takes the shape of a trapezium of which the height (*h*) was accurately measured with the microscope digital imaging tool. Only scratches of which the trace was perpendicular to the interface with a tolerance ±3 deg are considered (see Figure 2). The same criterion was used for scratches crossing the interface between the metal matrix and a porosity, labeled as “air” in Figure 6 and Table 3. The magnification of 15,000–20,000 was always as large as possible, to fit the complete trapezium in the image. The results of these measurements (minimum 5 measurements per type of interface) are plotted in Figure 6, which exhibits on the x-axis the applied load and on the y-axis the trapezium height (*h*). The plot of Figure 6 presents also the values of Δ*h*_1_ and Δ*h*_2_, whereby Δ*h*_1_ is the *h* difference observed between LP/NSG interfaces (averaged for all loads) and LP/air interfaces (Δ*h*_1_ = 1.03 μm) in the AC sample; while Δ*h*_2_ is the difference in *h* observed between F/SG (averaged for all loads) and F/NSG interfaces (averaged for all loads) in the HT samples (Δ*h*_2_ = 0.619 μm). 

## 4. Discussion

In the present experiment, four types of solid/solid interfaces are observed. In the AC sample, almost all interfaces are between lamellar pearlite and smooth graphite (LP/NSG) (the scatter in the data of Figure 6 is probably associated with sample preparation and material strength), whereas in the heat-treated samples, three types of interfaces can be discerned: (i) between ferrite and spiky graphite (F/SG), (ii) between ferrite and non-spiky graphite (F/NSG), and (iii) between lamellar pearlite and non-spiky graphite (LP/NSG). Additionally, also the interfaces have to be considered between the metal solid and the open atmosphere (air), which gives rise to a lamellar pearlite–air (LP/air) interface in the AC materials and a ferrite–air (F/air) interface in the HT samples. In Table 3, the values of the trapezium heights (*h*) and differences in the hardness (∆H), associated with each of these interfaces, are listed. 

**Figure 7:**
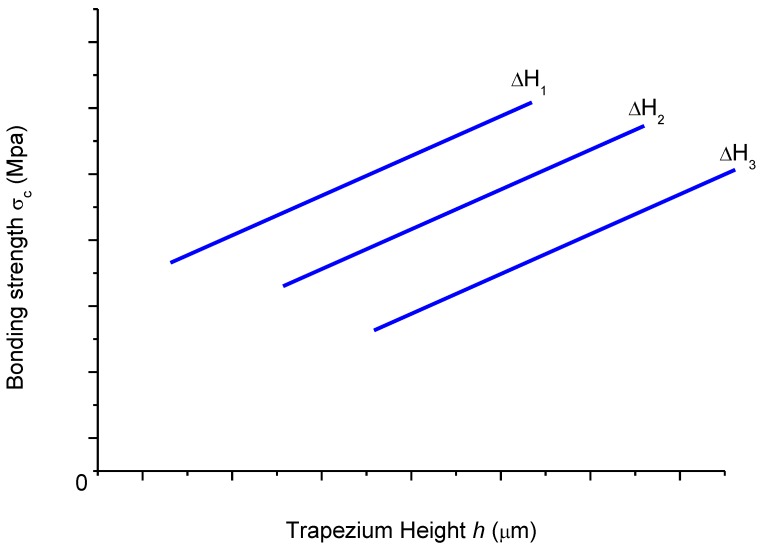
Schematic of the ascending correspondence between trapezium height *h* and bonding strength σ_c_, for a given value of hardness difference between the two phases. Actual data points of this correspondence could not be obtained here, given the lack of quantitative data of σ_c_.

An advanced theoretical model, requiring sophisticated finite element simulations, should be developed to describe the precise nature of the relation expressed in Equation 1. Pending such a theory, it is logical to assume, however, that there is an ascending relation between *h* and σ_c_ for constant values of ΔH as shown in the schematic plot of Figure 7, which can be derived by considering the limiting case of a delaminated interface, where evidently *h* → 0. The assumption is applied in the HT samples with ferrite matrix, where an increase of *h* was observed for the same ΔH when graphite was transformed from NSG to SG. It is not evident, however, to assert the relation between *h* and ΔH for constant values of σ_c_. One might reasonably assume that for a given bond strength σ_c_, ΔH affects the difference in width between the parallel sides of the trapezium. However, it is not possible to ascertain a definite positive or negative correlation between the height *h* of the trapezium and ΔH. Therefore, in the present study, only interfaces will be compared for which ΔH is constant. It is equally impossible to fill the conceptual graph of Figure 7 with real data points, as it is impossible to directly measure the bond strength σ_c_.

As the hardness of the graphite is much lower than the hardness of the metal matrix (cf. Table 3), it can be observed that the scratch widens up when the indenter crosses from the metal to the graphite phase (see Figure 5). Assuming that the bonding strength σ_c_ = 0, such as, e.g., would be the case for a fully delaminated graphite particle, it obviously would imply that *h* is very low, as this case is similar to the scratch approaching the pore edge of the metal sample. The data of Figure 5 reveal that under this circumstance, *h* < 1 μm, for the HT sample. It also implies that with increasing bonding strength σ_c_, the parameter *h* will increase, as the stress interaction between the two phases is amplified and distributed along the interface. Therefore, under the given experimental conditions, for a constant difference in hardness (∆H) between two phases, it may be interpreted that there is an increasing relation between *h* and σ_c_. 

**Figure 8:**
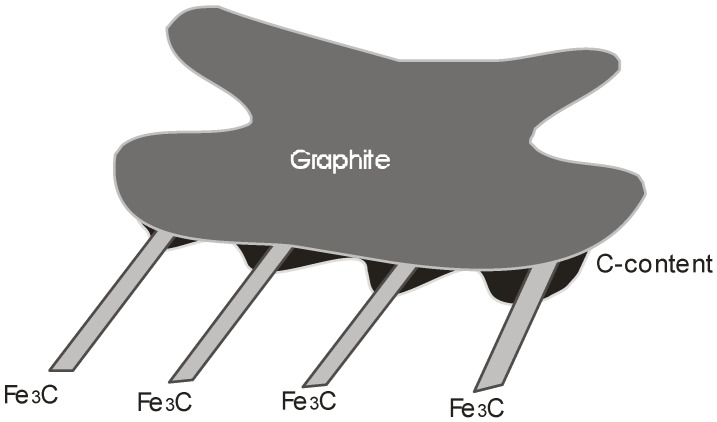
Schematic representation of the dissolving cementite lamella, producing a graphite–metal interface with spiky morphology.

From the data of Table 3, it is found that the average *h* associated with the F/SG interface is 42% larger than the average *h* associated with F/NSG (same ΔH), which indicates a stronger bonding strength between spiky graphite and ferrite, as compared with the interface between non-spiky graphite and ferrite. Given the morphology of the interface, it could be assumed that this bonding strength is of a mechanical nature [26,27,28,29]. Evidently, the emerging morphology itself, is a consequence of the diffusional mechanism of C dissolving from the cementite Fe_3_C (Fe_3_C → Fe + 3C) and migrating to the graphite interface [30], where intrusions and extrusions are possibly formed as a result of the Fe_3_C lamella intersecting with the graphite particles and, at such intersection points, the Fe_3_C dissolution will occur preferentially [31]; see Figure 8. The microcrystalline spikes measured by TEM (using selected area diffraction) by several authors [32,33,34] confirmed that the decomposing cementite plates gradually dissolved at the M–G interface. This model also implies that the spiky character of the graphite–ferrite interface is of a transient nature, because with increasing annealing time, the carbon concentration will gradually be homogenized at the M–G interface. It is surmised here that the increased mechanical bond at the spiky ferrite–graphite interface could enhance the fatigue lifetime of CGI, as reported by Ghodrat et al. [11], by delaying the delamination at the M–G interface, and thus delaying the nucleation rate of voids.

Table 3 shows that the h value, associated with the LP/NSG interface in the AC samples, attains a level of 4.14 μm, which is significantly higher than the h values observed for the heat-treated samples. However, it cannot be derived from this that the LP/G interface in the AC material has a stronger bonding than the interfaces of the HT samples, because the ∆H between lamellar pearlite and graphite is different from ∆H values observed in the heat-treated samples (cf. Table 3).

## 5. Conclusions

In this paper, a method is proposed to differentiate the bonding strength (σ_c_) between various types of interfaces. In particular, it was applied to the various metal-graphite interfaces in CGI, changing from smooth to spiky by applying an isothermal annealing treatment for 60 h at 700 °C. The results obtained in this study showed that the interface strength between ferrite and smooth graphite is lower than the strength of the interface between ferrite and spiky graphite. This conclusion is relevant in view of the fact that it was proven earlier that mechanical properties of CGI samples were improved significantly by an appropriate annealing treatment. A precise quantitative determination of the interface strengthneeds a detailed micro-mechanical model of the scratch delamination process.

## Figures and Tables

**Table 1:** Microstructural characteristics of compacted graphite (CG) and spheroidal graphite particles (SG).

Dimension	Size	
CG width	4.14 μm (±2.01)	
CG maximum length	45 μm (±25)	
CG aspect ratio	3.01 μm (±2.1)	
SG diameter	18 μm (±5)	
SG nodularity (area fraction of nodular graphite particles)		15.6%

**Table 2:** Chemical composition of samples (weight % and standard deviation). The reference composition is given in [1].

wt %	Si	Cr	Mn	Ti	Cu	Mg	C
**AC**	1.25 (0.33)	0.09 (0.05)	0.45 (0.13)	0.07 (0.01)	0.9 (0.22)	0.08 (0.09)	-
**HT LP**	0.95 (0.13)	0.14 (0.18)	0.73 (0.19)	0.07 (0.05)	0.39 (0.12)	0.08 (0.05)	-
**HT F**	1.65 (0.13)	0.09 (0.01)	0.35 (0.01)	0.04 (0.008)	1.2 (0.87)	0.08 (0.06)	-
**Ref [1]**	1.9–2.2	<0.1	0.15–0.4	<0.015	0.75–0.95	0.004–0.01	3.6–3.9

**Table 3:** Trapezium height *h* (μm) and HVN hardness difference ΔH at different type of interfaces for HT and AC samples. SG = spheroidal graphite, NSG = non-spiky graphite, LP = lamellar pearlite, and F = ferrite. Values between parentheses are standard deviations.

Phase	HT		AC
	LP	F	LP
	*h* (μm) ΔH (HVN)	*h* (μm) ΔH (HVN)	*h* (μm) ΔH (HVN)
SG	-	2.13 (0.8) 103 (3.8)	-
NSG	2.15 (0.7) 204 (4.5)	1.5 (0.5) 103 (6.4)	4.14 (1.4) 226 (7.2)
Air	-	0.911 (0.01) 133 (2.7)	3.05 (0.35) 256 (5.5)

## Abbreviations

The following abbreviations are used in this manuscript:

CGI	compacted graphite iron
CG	compacted graphite
SI	spheroidal graphite
AC	as cast
HT	heat treated
TMF	thermomechanical fatigue
FE	finite element
M–G	metal–graphite
ε	strain
*h*	trapezium height
ΔH	difference in materials‘ hardness
σ_c_	critical stress normal to the M–G interface
LP	lamellar pearlite
SG	spiky graphite
NSG	non-spiky graphite
F	ferrite
STDEV	standard deviation
